# Author Correction: Predicting fertility from sperm motility landscapes

**DOI:** 10.1038/s42003-022-04060-x

**Published:** 2022-10-13

**Authors:** Pol Fernández-López, Joan Garriga, Isabel Casas, Marc Yeste, Frederic Bartumeus

**Affiliations:** 1 Theoretical and Computational Ecology Group, Centre d’Estudis Avançats de Blanes (CEAB-CSIC), Cala Sant Francesc, 14, 17300 Blanes, Spain; 2 Biotechnology of Animal and Human Reproduction (TechnoSperm), Institute of Food and Agricultural Technology, University of Girona, 17003 Girona, Spain; 3 Unit of Cell Biology, Department of Biology, Faculty of Sciences, University of Girona, 17003 Girona, Spain; 4 Institució Catalana de Recerca i Estudis Avançats, ICREA, Passeig Lluís Companys, 23, 08010 Barcelona, Spain; 5 Centre de Recerca Ecològica i Aplicacions Forestals (CREAF), Cerdanyola del Vallès, 08193 Barcelona, Spain

**Keywords:** Functional clustering, Bayesian inference

Correction to: *Communications Biology* 10.1038/s42003-022-03954-0, published online 28 September 2022.

In this article the affiliation Theoretical and Computational Ecology Group, Centre d’Estudis Avançats de Blanes (CEAB-CSIC), Cala Sant Francesc, 14, 17300 Blanes, Spain for Frederic Bartumeus was missing. The original article has been corrected.

