# Supplementary material for: Predicting fertility from sperm motility landscapes
Source: Commun Biol. 2022 Sep 28;5:1027. doi: 10.1038/s42003-022-03954-0 (PMC9519750; doi:10.1038/s42003-022-03954-0)
Supplement: Supplementary file 2 — Supplementary Material [file 42003_2022_3954_MOESM2_ESM.pdf]

Predicting fertility from sperm motility landscapes

Supplementary Material

Pol Fernández-López[1], Joan Garriga[1], Isabel Casas[2,3], Marc Yeste[2,3,4], and Frederic Bartumeus[4,5,*]

[1]Theoretical and Computational Ecology Group, Centre d'Estudis Avançats de Blanes (CEAB-CSIC), Cala Sant Francesc, 14, 17300 Blanes, Spain

[2]Biotechnology of Animal and Human Reproduction (TechnoSperm), Institute of Food and Agricultural Technology, University of Girona, 17003 Girona, Spain 2

[3]Unit of Cell Biology, Department of Biology, Faculty of Sciences, University of Girona, 17003 Girona, Spain

[4]Institució Catalana de Recerca, ICREA, Passeig Lluís Companys, 23, 08010 Barcelona, Spain

[5]Centre de Recerca Ecològica i Aplicacions Forestals (CREAF), Cerdanyola del Vallès, 08193 Barcelona, Spain

[*]Corresponding author: Frederic Bartumeus, fbartu@ceab.csic.es

Supplementary Note 1

We provide here (Fig. S1) a schematic representation of the four sperm motility variables used in the computer-aided sperm analysis (CASA) that we also used in our analysis. This should help in the interpretation and understanding of the results reported in this manuscript (Fig. 3, Fig. 5, and Fig. S2.)

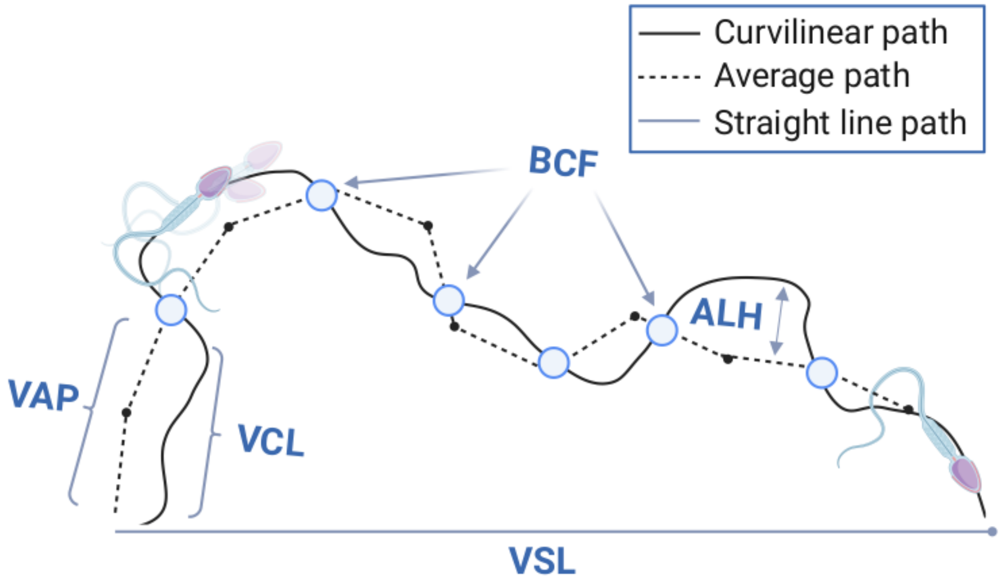

Figure S1: Representation of the main sperm motility variables of computer-aided sperm analysis (CASA). A fictional sperm trajectory is shown, and the key motility measures of CASA (that we used as input features in our analysis) are depicted. **VCL** = curvilinear velocity, in μm/s: velocity measured with the actual path of the spermatozoa. **VSL** = straight-line velocity, in μm/s: velocity measured from the initial to the last point of the trajectory. **VAP** = average path velocity, in μm/s: velocity measure with an averaged trajectory. **ALH** = amplitude of lateral head displacement, in μm: distance from the position of the head to the average trajectory. **BCF** = beat cross frequency, in Hz: inverse of the number of times that the head crosses the average trajectory. **STR** = straightness, in %: $100 \times VSL/VAP$, measures the straightness of the trajectory (not shown). **LIN** = linearity, in %: $100 \times VSL/VCL$, measures the linearity of the trajectory (not shown). **WOB** = wobble, in %: $100 \times VAP/VCL$, measures the wobble of the trajectory (not shown). Created with BioRender.com, and published under the corresponding license permissions.

Supplementary Note 2

The sperm motility landscape can be visualized and inspected in different ways (Figures S2, S3 and S4).

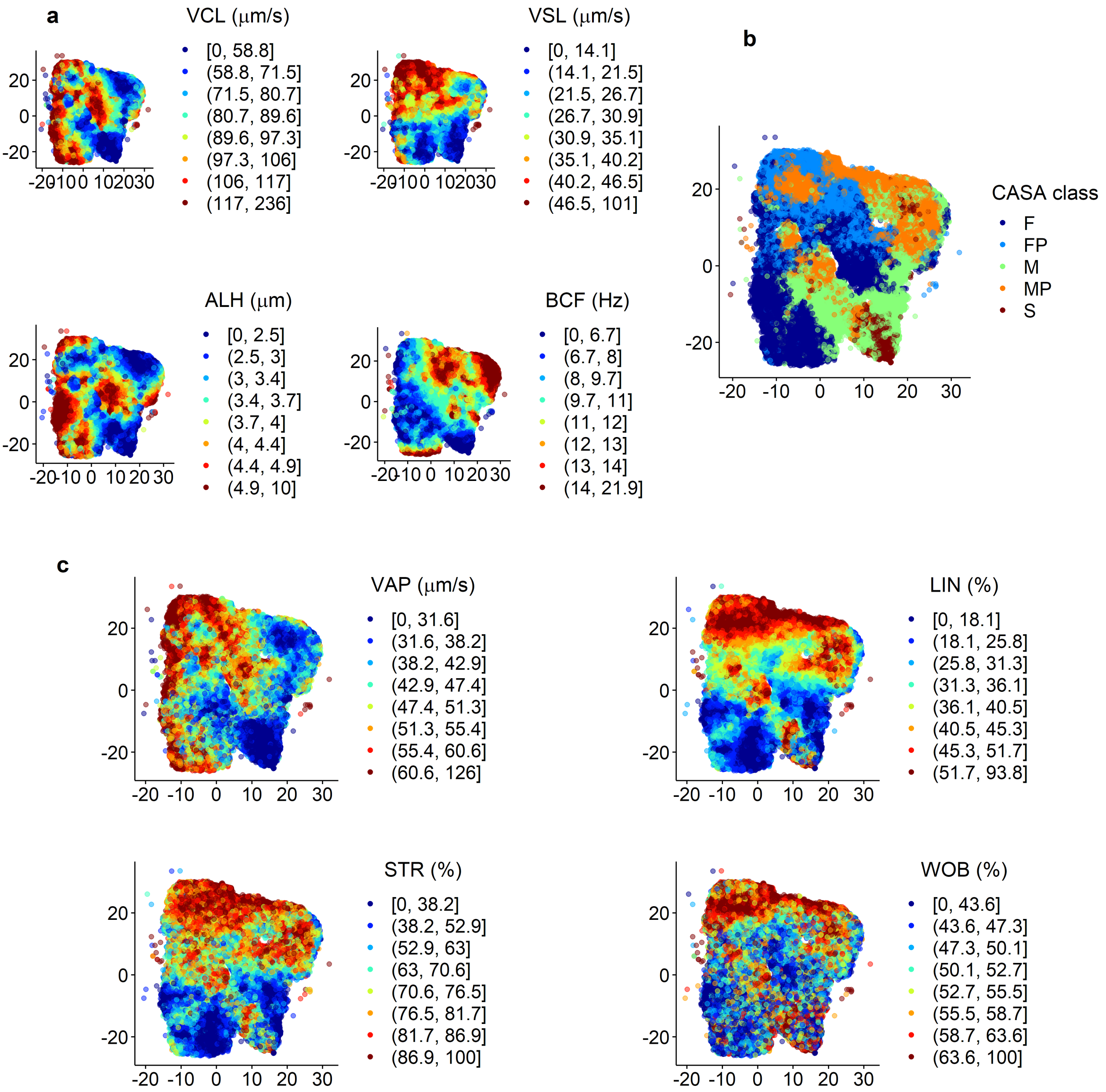

Figure S2: Sperm motility input features included in the t-SNE, and comparison to CASA classification. **a** Quantile heatmap of the features used in the t-SNE (VCL, curvilinear velocity, VSL, straight line velocity, ALH, lateral amplitude of the head displacement and BCF, beat-cross frequency). Colors represent an interval of values (eight percentiles, from 0-12.5 to 87.5-100) of each variable amended for a gradient visualization. **b** Mapping of the five-class classification from the CASA system (F, fast sperm; FP, fast progressive sperm; M, medium-speed sperm; MP, medium-speed progressive sperm; S, slow sperm). Note the high correspondence between VAP and the different classes. **c** Quantile map of external motility variables related to the four fundamental sperm motility features used in t-SNE (VAP, average path velocity; LIN, linearity 100 x VSL/VCL; STR, straightness 100 x VSL/VAP, wobble, WOB 100 x VAP/VCL). Color gradient represents an interval of values (eight percentiles, from 0-12.5 to 87.5-100) of each variable from large (hot) to low (cold) values (colors).

For instance, by displaying the values of the different input features (variables) used to construct the landscape one can assess the biological meaning (in terms of motility) of different regions of the landscape. In Fig. S2a we show the four sperm motility parameter values used to build the landscape: curvilinear velocity (VCL), straight-line velocity (VSL), amplitude of lateral head displacement (ALH) and beat-cross frequency (BCF). It is plainly visible how VCL and ALH are closely related, as there is high correspondence between the two quantile heatmaps.

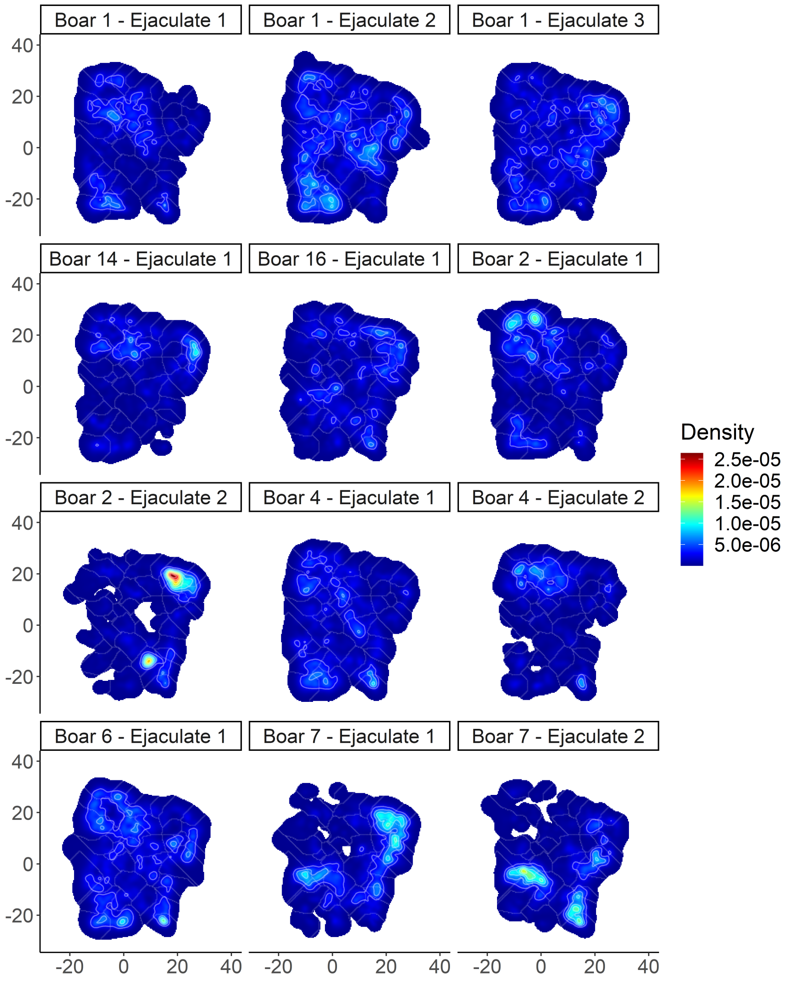

Figure S3: Visualization of the variability across boars and ejaculates. Note that different boars and ejaculates show different sperm motility prevalence, and some ejaculates are highly concentrated in specific regions, whilst others are widespread along the whole landscape. The scale of density is common for all figures. The contours are depicted to help interpret the density, and the clear lines are the limits to the clusters, as shown in Figure 1.

We can also map external variables (not used in the analysis), such as other relevant information associated to sperm motility in the landscape (Fig.S2b-c). For example, we mapped the

five sperm motility categories obtained with standard CASA algorithms (Fig. S2b). Note its correspondence with VCL and VAP values, showing that CASA classification is mainly driven by sperm speed (i.e. VAP). In Fig. S2c, we also mapped other variables like the average path velocity (VAP), and specific motility quotients obtained from the four basic sperm motility features used (VCL, VSL, ALH and BCF). Note that there was a tight correspondence between VCL and VAP, and a good correlation between LIN, STR and VSL, despite in this latter case, some differences were also observed.

To visually inspect how inter- and intra- (ejaculates) boar variability shows up in the overall sperm motility landscape, we specifically visualize sperm motility landscapes per boar and ejaculate (Fig. S3). Under the assumption that boar and ejaculate variability are low, one would expect single boar/ejaculates to be spread all over the landscape. However, sperm from different boars, and even from different ejaculates, occupied distinct regions of the map (Fig. S3). Indeed, some ejaculates had a great variety of behaviours scattered over the landscape (e,g. boar 1 ejaculates 1, 2 and 3). Other ejaculates had a narrower spectrum of behaviours, with sperm concentrated in specific regions of the landscape (e.g. boar 2, ejaculate 2; boar 4, ejaculate 2; boar 7, ejaculate 2). It is worth noting that ejaculates from a particular boar could also display very different behaviours (e.g. boar 2, ejaculates 1 and 2).

Supplementary Note 3

The landscape can be discretized into clusters, by different statistical methods. We used a watershed algorithm, and merged the resulting partitions into a total of 11 clusters (see Results and Methods). Here (Fig. S4), we present the features of each cluster, and order them according to their effect on fertility (negative to positive, from left to right).

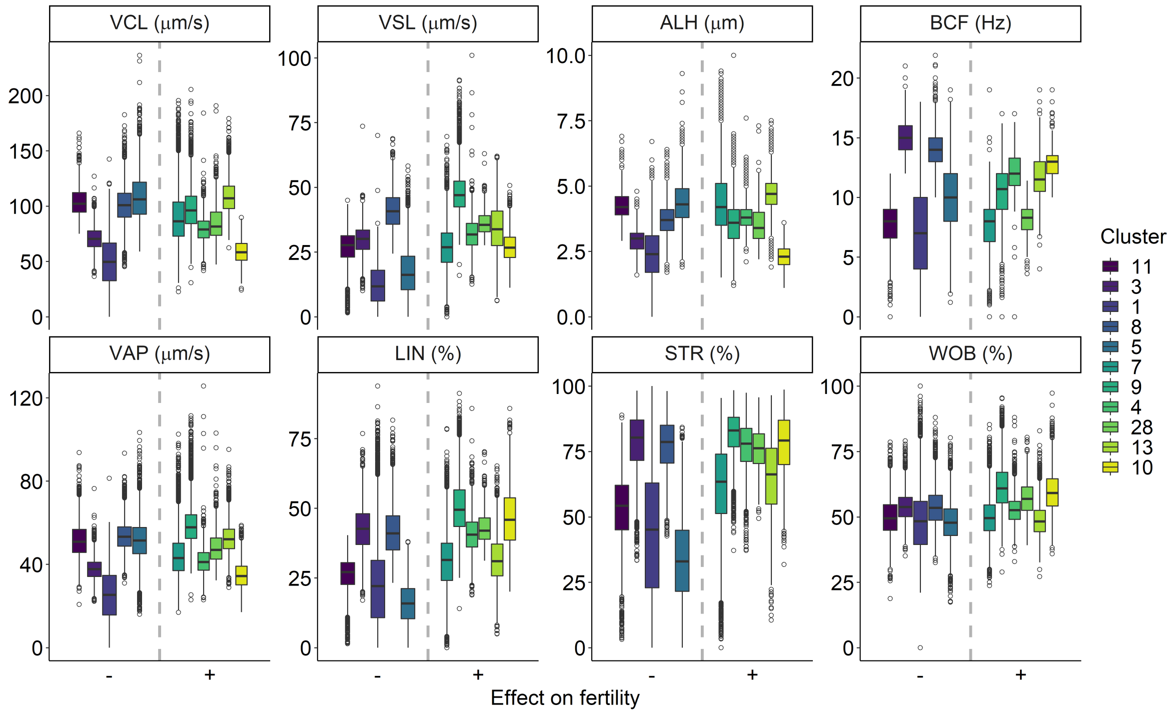

Figure S4: Sperm motility features across landscape clusters and their effect on boar fertility. Boxplots of the sperm motility features (VCL, curvilinear velocity; VSL, straight line velocity; ALH lateral amplitude of head displacement; BCF, beat-cross frequency; VAP, average path velocity; LIN, linearity; STR, straightness; WOB, wobble) in the 11 clusters that conformed the motility landscape in our most predictive fertility model (model M3). Clusters were ordered from left to right, in consonance with their effect on fertility (quantified from negative to positive, according to the medians of the coefficients resulting from the fertility model M3), and colored accordingly.

Supplementary Note 4

Perplexity is a neighbouring parameter setting the scale where datapoint similarities in the t-SNE algorithm are assessed. This parameter modulates the trade-off between emphasizing the local or the global structure of the 2D embedded landscape (see Van der Maaten 2008 for details[1]). At low perplexities, fewer points are considered as neighbours and similarities are compared only across small subsets of neighbouring points, hence, a greater focus on local landscape structures is achieved. Opposed to that, high perplexities include large number of neighbours, so that similarity metrics is applied over broader neighbourhood ranges, which is better for a representation that reflects the global structure of data.

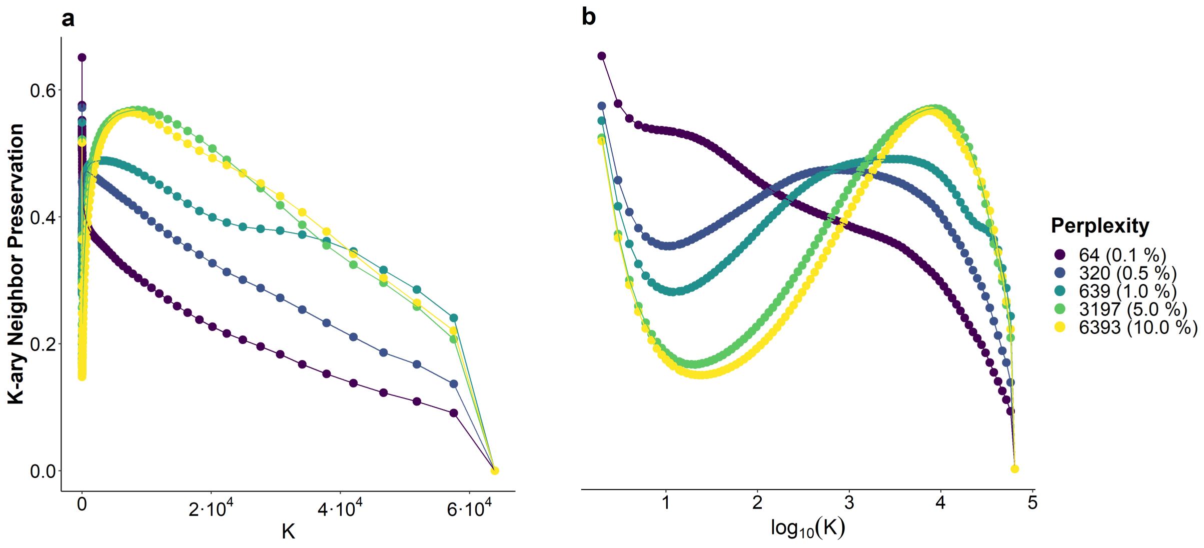

Figure S5: K-ary neighbour preservation (KNP) as a quality measure for 2D embeddings obtained through t-SNE methods. By means of KNP, we explored how different perplexities in the t-SNE algorithm generate landscapes of different quality. We show the correspondence high-dimensional vs. low-dimensional space for different neighbourhood sizes (K) across different perplexities; from 64 to 6,393, representing from 0.1% to 10% dataset size, respectively. **a** KNP for different neighbourhood sizes, as a measure of global structure preservation. **b** KNP for different neighbourhood sizes in logarithmic scale, as a measure of local structure preservation. See also Garriga & Bartumeus, 2021[2]).

To comprehensively quantify this effect, we measured the local and global structure preservation across a range of perplexities as suggested in Garriga & Bartumeus, 2021[2]. We scanned

different perplexities, ranging from a 0.1% to a 10% of the size of the dataset, and compared the quality of the obtained 2D embeddings (Fig. S5). A Barnes-Hut implementation of the t-SNE[3] algorithm was run for each perplexity, using the bigMap R package[4](available in https://github.com/jgarriga65/bigMap, version 4.5.3). Each of these t-SNE outputs were assessed in terms of k-ary neighbour preservation (KNP), a consensus measure for embeddings resulting from dimensionality reduction methods[5–8]. Specifically, KNP is a quality measure that compares neighbouring points between high-dimensional and low-dimensional spaces[8]. Following Garriga & Bartumeus, 2021[2], the neighbourhood size K is expressed both linearly (K) (Fig. S5a) and as $log_{10}(K)$ (Fig. S5b), depicting the quality of the global structure and the local structure, respectively. The area under the curve of both functions was calculated to obtain a quantification of global and local structure qualities across all of the embeddings (Table S1).

Table S1: K-ary neighbourhood preservation. The correspondence between the original data (high-dimensional) and the low dimensional space resulting from t-SNE measures the quality of the embedding. The area under the curve (AUC) of the quality across different neighbourhood sizes (linAUC), and the AUC of its log-linear representation (logAUC) were used to assess the global structure and the local structure (logAUC), respectively. These were measured over a range of perplexities from 64 to 6,393 (0.1% to 10% of the size of the dataset), revealing that the best balance (ratio closest to 1, presented as absolute value of the difference $1 - Ratio$) between global and local structure was obtained at 1% (639) perplexity.

**Perplexity**	**64**	**320**	**639**	**3197**	**6393**
**linAUC**	0.179	0.253	0.336	0.366	0.368
**logAUC**	0.462	0.409	0.380	0.312	0.293
**Ratio**	0.387	0.620	0.885	1.175	1.254
$\|\mathbf{1 - Ratio}\|$	0.613	0.380	0.115	0.175	0.254

A progressive shift from best local structure preservation (perplexity = 64, 0.1 %) to best global structure preservation (perplexity = 6,393, 10 %) is observable in Table S1, where K-AUC curves increased and $log_{10}(K)$-AUC curves decreased as we moved from low to large perplexities. However, the best trade-off between global and local structure (ratio closer to 1) is achieved at 1 % perplexity (639). Consequently, perplexity was set at 639 to generate the landscape for further analysis.

Supplementary Note 5

In order to assess the robustness of our results, we applied the same procedure of analysis (see Methods: Sperm motility landscape) using two additional methods for data dimension reduction: (i) the Fast-Fourier Interpolation-based t-SNE (FIt-SNE)[9], and the Uniform Manifold Approximation and Projection (UMAP)[10].

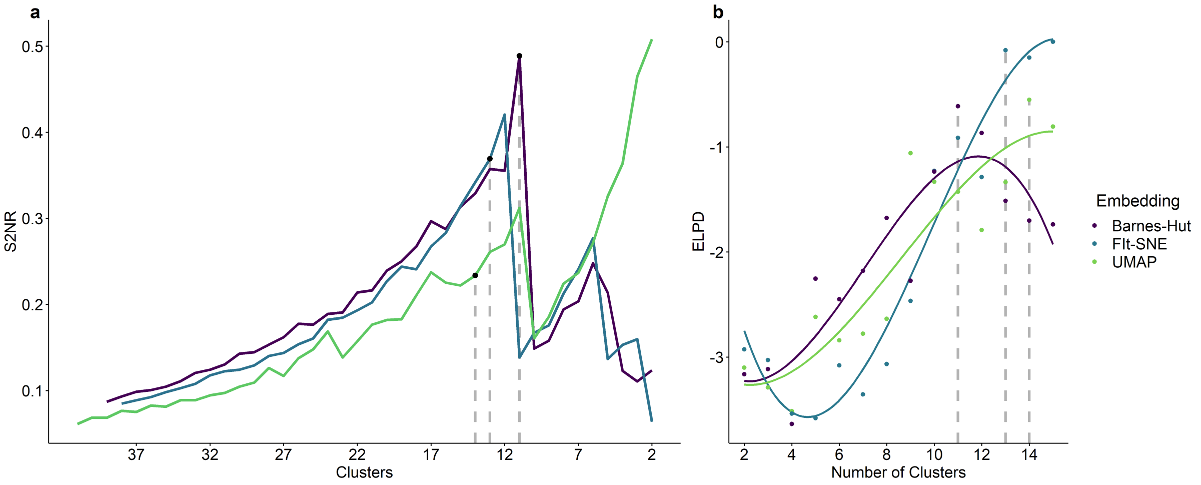

Figure S6: Comparison of landscape merging scales for different data dimension reduction algorithms: Barnes-Hut t-SNE, Fast-Fourier Interpolation-based t-SNE (FIt-SNE) and Uniform Manifold Approximation and Projection (UMAP). **a** Signal to noise ratio (S2NR) as a measure of information gain along the process of merging the clusters in the landscape. The vertical dotted lines represent the best models selected for algorithm (Barnes-Hut, FIt-SNE and UMAP). **b** Approximate leave-one-out cross-validation, used to assess model performance (loo R package). The ELPD corresponds to the expected log pointwise predictive density, as a measure of predictive capability of the model, being ELPD = 0 the best performing model. Models of different landscape configurations (merging scales) are compared across the different dimension reduction methods; the Barnes-Hut t-SNE (deep purple), the FIt-SNE (dark green) and the UMAP (light green). The models chosen for comparison (i.e. best models according to S2NR and ELPD criteria) are highlighted with a vertical dotted line.

A FIt-SNE with perplexity 1% (639), and a UMAP with a number of neighbors set to 639 were run. The KDE computed for both embeddings was run with the same perplexity (639). The metrics involving the merging (Fig. S6) and the predictions of the model (Fig. S7) were both assessed and compared between FIt-SNE, UMAP and Barnes-Hut t-SNE. As shown in Figure S6a, the initial number of clusters was similar in all embeddings (39 for Barnes-Hut, 38 for FIt-SNE and 41 for UMAP), and the merging process led to a similar hierarchical organization of the landscape. The three embeddings showed a critical drop of information after

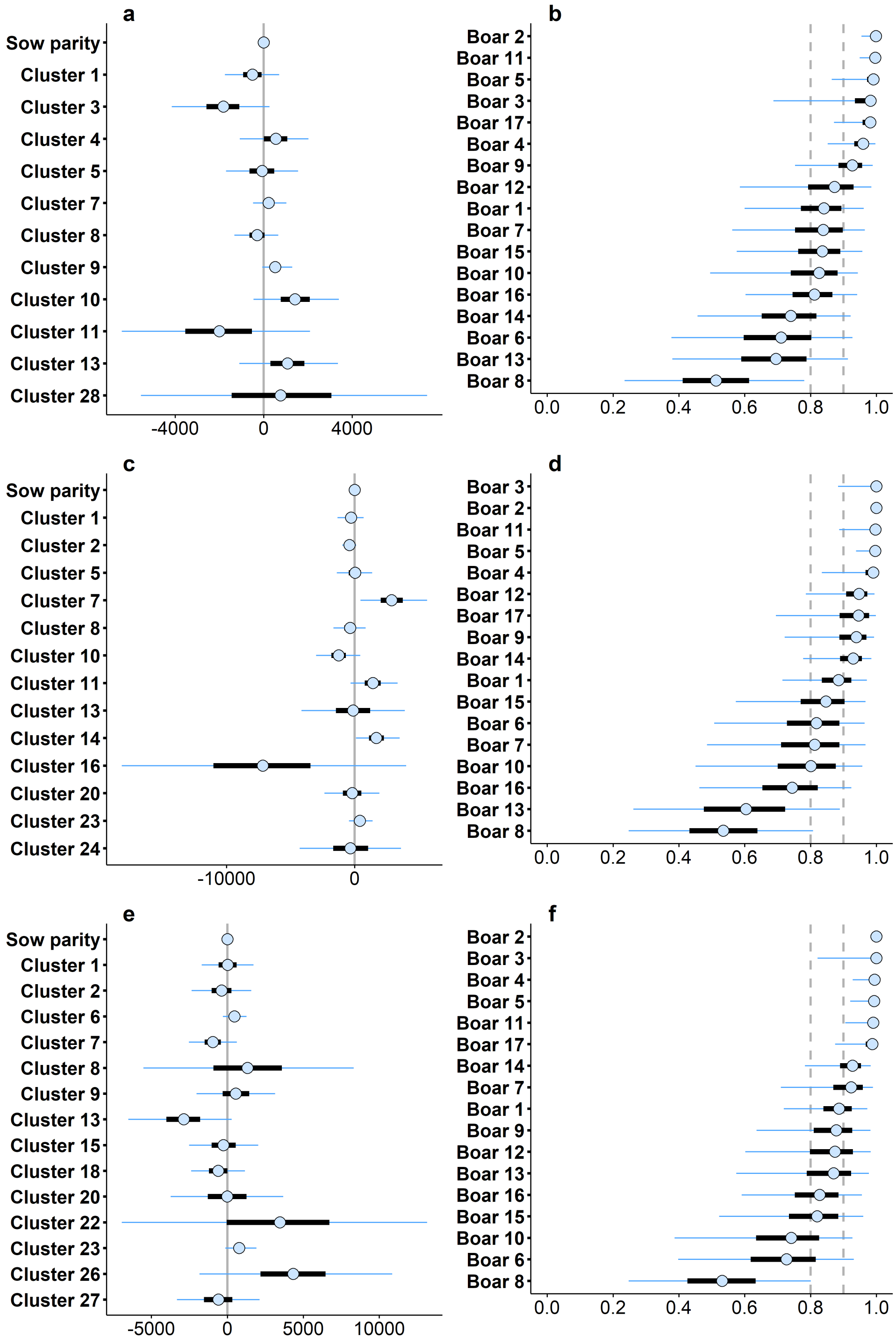

Figure S7: Comparison of the model outputs for each dimension reduction algorithm tested (Barnes-Hut t-SNE, FIt-SNE and UMAP). Left panels: Coefficients of the sperm motility features for the models obtained with Barnes-Hut t-SNE **a**, FIt-SNE **c** and UMAP **e**. The medians of the coefficients are represented as clear dots, the 50% C.I. as a thick black line, and the 95% C.I. as a blue thin line. Right panels: Predicted fertility outcomes per boar in the models obtained for Barnes-Hut t-SNE **b**, FIt-SNE **d** and UMAP **f**. The vertical dotted lines represent threshold at 0.8 and 0.9, corresponding with a subjective categorisation of the boars. The medians of the predictions are represented as clear dots, the 50% C.I. as a thick black line, and the 95% C.I. as a blue thin line.

the 11 cluster organization, indicating the existence of an optimal arrangement of the landscape variability. Whilst this scale represented the highest S2NR peak in Barnes-Hut and FIt-SNE, UMAP exhibited an uptrend beyond this point. Likely, this was due to a lack of structure in the UMAP embedding with less than 11 clusters (higher variability within clusters than between clusters).

When comparing the predictive density of the models (ELPD), a similar pattern appeared in all embeddings (Fig. S6b). Coarser configurations of the landscape (i.e. two to eight clusters) involved models with worse accuracies than landscapes conformed by nine to 15 clusters. Specifically, the best models were obtained with 11 clusters the Barnes-Hut embedding, 13 to 15 clusters for FIt-SNE, and 14 to 15 clusters for UMAP. The FIt-SNE landscape with 13 clusters showed the largest information (S2NR) and better interpretability (smaller number of clusters). The UMAP landscape with 14 clusters showed the best ELPD, and good enough S2NR. Accordingly, we compared these two models (FIt-SNE with 13 clusters, and UMAP with 14 clusters) to the Barnes-Hut model with 11 clusters.

The qualitative meaning of the clusters (positive or negative correlation to fertility), and the scales of the coefficients (orders of magnitude) of the different models were found to be comparable (Fig. S7a,c,e). Nevertheless, FIt-SNE coefficients exhibited, in general, smaller credible intervals (with less uncertainty), and more clusters near the value of 0 (less impact on fertility) compared to the Barnes-Hut model. The semantics of the clusters were similar in the three models: clusters with positive influence in fertility accounted for a higher VSL, LIN and STR, compared to clusters with negative correlation to fertility.

The predictions obtained with the models (Fig. S7b,d,f) yielded a similar classification of boars. Both the estimated fertility ratios and their ranking were analogous (Kendall correlations: $cor(BH|FIT) = 0.779$, pvalue $= 1.3 \times 10^{-5}$; $cor(BH|UMAP) = 0.647$, pvalue $= 2.9 \times 10^{-4}$; $cor(FIT|UMAP) = 0.662$, pvalue $= 2.1 \times 10^{-4}$). Additionally, it is evident that the models make more accurate predictions (less uncertain) with boars showing high fertility

rates. However, they reveal the presence of 3 qualitative groups: (i) boars with high fertility rate estimates (above 0.9) (positions 1 to 7 in Barnes-Hut model; 1 to 9 in FIt-SNE model; 1 to 8 in UMAP model, e.g. Boar 2,3,4,5,11,17). Note that in the FIt-SNE and UMAP models this group could be even subdivided in two groups, with the first five (six in case of UMAP) boars in the rank showing very high (around 0.99) and accurate estimates of fertility rates; (ii) boars with intermediate fertility rates (between 0.8 and 0.9) and higher uncertainty (positions 8 to 13 in Barnes-Hut model; 10 to 14 in FIt-SNE model; 9 to 14 in UMAP model, e.g. Boars 1,15); (iii) boars with low fertility rates (below 0.8) and high uncertainty in the estimates (positions 14 to 17 in Barnes-Hut model; 15 to 17 in FIt-SNE and UMAP models, e.g. Boar 8). The above categorisation is indeed qualitative and somewhat arbitrary, based on the staircase-ranked pattern of the fertility medians and the amplitude of credible interval percentiles, with the only purpose to highlight robustness in our boar rank classification. Note that there are some differences between the three models. However, the models share more similarities when compared pairwise (i.e. Barnes-Hut t-SNE is similar to FIt-SNE and UMAP, but the three models compared all at once have less consistent classification of boars).

Supplementary References

1. Van Der Maaten, L. & Hinton, G. Visualizing Data using t-SNE. *Journal of Machine Learning Research* **9,** 2579–2605. ISSN: 1533-7928 (2008).

2. Garriga, J. & Bartumeus, F. Towards a comprehensive visualization of structure in data. Preprint at: `https://arxiv.org/abs/2111.15506` (2021).

3. Van der Maaten, L. Barnes-Hut-SNE. Preprint at: `https://arxiv.org/abs/1301.3342` (2013).

4. Garriga, J. & Bartumeus, F. *Big data mapping with parallelized t-SNE* version v4.5.3. Zenodo, 2021. `https://doi.org/10.5281/zenodo.5506846`.

5. France, S. & Carroll, D. *Development of an agreement metric based upon the RAND index for the evaluation of dimensionality reduction techniques, with applications to mapping customer data* 499–517. ISBN: 9783540734987 (Springer Verlag, 2007).

6. Venna, J., Peltonen, J., Nybo, K., Aidos, H. & Kaski, S. Information Retrieval Perspective to Nonlinear Dimensionality Reduction for Data Visualization. *Journal of Machine Learning Research* **11,** 451–490 (2010).

7. Chen, L. & Buja, A. Local multidimensional scaling for nonlinear dimension reduction, graph drawing, and proximity analysis. *Journal of the American Statistical Association* **104,** 209–219. ISSN: 01621459 (2009).

8. Lee, J., Peluffo, D. & Verleysen, M. Multi-scale similarities in stochastic neighbour embedding: Reducing dimensionality while preserving both local and global structure. *Neurocomputing* **169** (2015).

9. Linderman, G. C., Rachh, M., Hoskins, J. G., Steinerberger, S. & Kluger, Y. Fast interpolation-based t-SNE for improved visualization of single-cell RNA-seq data. *Nature Methods* **16,** 243–245. ISSN: 15487105 (2019).

10. McInnes, L., Healy, J. & Melville, J. UMAP: Uniform Manifold Approximation and Projection for Dimension Reduction. Preprint at: `https://arxiv.org/abs/1802.03426` (2018).
