# Supplementary material for: Predicting fertility from sperm motility landscapes
Source: Commun Biol. 2022 Sep 28;5:1027. doi: 10.1038/s42003-022-03954-0 (PMC9519750; doi:10.1038/s42003-022-03954-0)
Supplement: Supplementary file 3 — Description of Additional Supplementary Files [file 42003_2022_3954_MOESM3_ESM.pdf]

Description of Additional Supplementary Files

File name: Supplementary Data
Description: Includes the raw data allowing to reproduce the MS results, and the raw data used in each figure.

File name: Supplementary Code
Description: The source code of data analysis Page 6 of 12 and figures conception.
