# Supplementary material for: Predicting fertility from sperm motility landscapes
Source: Commun Biol. 2022 Sep 28;5:1027. doi: 10.1038/s42003-022-03954-0 (PMC9519750; doi:10.1038/s42003-022-03954-0)
Supplement: Supplementary file 6 — Reporting Summary [file 42003_2022_3954_MOESM6_ESM.pdf]

nature portfolio

Corresponding author(s): Frederic Bartumeus

Last updated by author(s): Aug 23, 2022

Reporting Summary

Nature Portfolio wishes to improve the reproducibility of the work that we publish. This form provides structure for consistency and transparency in reporting. For further information on Nature Portfolio policies, see our Editorial Policies and the Editorial Policy Checklist.

Statistics

For all statistical analyses, confirm that the following items are present in the figure legend, table legend, main text, or Methods section.

n/a	Confirmed	
☐	☒	The exact sample size (*n*) for each experimental group/condition, given as a discrete number and unit of measurement
☐	☒	A statement on whether measurements were taken from distinct samples or whether the same sample was measured repeatedly
☐	☒	The statistical test(s) used AND whether they are one- or two-sided<br>*Only common tests should be described solely by name; describe more complex techniques in the Methods section.*
☐	☒	A description of all covariates tested
☐	☒	A description of any assumptions or corrections, such as tests of normality and adjustment for multiple comparisons
☐	☒	A full description of the statistical parameters including central tendency (e.g. means) or other basic estimates (e.g. regression coefficient) AND variation (e.g. standard deviation) or associated estimates of uncertainty (e.g. confidence intervals)
☐	☒	For null hypothesis testing, the test statistic (e.g. *F*, *t*, *r*) with confidence intervals, effect sizes, degrees of freedom and *P* value noted<br>*Give P values as exact values whenever suitable.*
☐	☒	For Bayesian analysis, information on the choice of priors and Markov chain Monte Carlo settings
☐	☒	For hierarchical and complex designs, identification of the appropriate level for tests and full reporting of outcomes
☐	☒	Estimates of effect sizes (e.g. Cohen's *d*, Pearson's *r*), indicating how they were calculated

Our web collection on statistics for biologists contains articles on many of the points above.

Software and code

Policy information about availability of computer code

Data collection	Commercial software was used to process sperm motility videos: ISAS software (Integrated Sperm Analysis System V1.0; Proiser SL, Valencia, Spain).
Data analysis	Open source code was used for statistical analysis and result visualization:<br>For t-SNE based analysis, bigMap (version 4.5.3, available in https://doi.org/10.5281/zenodo.5506846 and https://github.com/jgarriga65/bigMap) and FIt-SNE (version 1.2.1, available in https://github.com/KlugerLab/FIt-SNE) R packages were used. For the UMAP embedding we used umap-learn (version 0.4.6) library in Python (version 3.6.8).<br>For Bayesian analysis, rstanarm R package (version 2.21.1, available in CRAN) was used.<br>Custom code was used for data processing and result visualization:<br>Code for data processing, figures and statistical analysis (parametrization, automatization, ...) are be available at https://github.com/Polfe94/sperm_move and zenodo (10.5281/zenodo.7015571).<br>All analysis performed in R used the version 4.0.2 of the software.

For manuscripts utilizing custom algorithms or software that are central to the research but not yet described in published literature, software must be made available to editors and reviewers. We strongly encourage code deposition in a community repository (e.g. GitHub). See the Nature Portfolio guidelines for submitting code & software for further information.

Data

Policy information about availability of data

All manuscripts must include a data availability statement. This statement should provide the following information, where applicable:

- Accession codes, unique identifiers, or web links for publicly available datasets
- A description of any restrictions on data availability
- For clinical datasets or third party data, please ensure that the statement adheres to our policy

Data is available in a Mendeley Data repository (DOI: 10.17632/jd38jhxpg6.3).

Field-specific reporting

Please select the one below that is the best fit for your research. If you are not sure, read the appropriate sections before making your selection.

☒ Life sciences ☐ Behavioural & social sciences ☐ Ecological, evolutionary & environmental sciences

For a reference copy of the document with all sections, see nature.com/documents/nr-reporting-summary-flat.pdf

Life sciences study design

All studies must disclose on these points even when the disclosure is negative.

Sample size	All data meeting the inclusion criteria (young age and semen collection during Spring period) was included in the study. A total of 36 ejaculates belonging to 17 animals (N = 17) were analyzed. Fertility data on farm animals is limited, specially given our restrictive criteria. Nevertheless, 17 animals is a reasonable sample size that can be representative of the young subset of the population, as one of the goals of the study was to characterize sperm variability. On top of that, multiple ejaculates from these animals were analyzed, increasing the amount of data analyzed in the study.
Data exclusions	Data exclusion criteria was age (animals older than 1 year and 30 days were excluded) and seasonality (data not comprised between March to June, Spring period, was excluded). This selection was established before any analysis were performed in the study, and its purpose was to limit uncontrolled external factors of variability.
Replication	All analysis (t-SNE, clusterization and Bayesian logistic regressions) were repeated with the same data-set, with variations in t-SNE method (Barnes-Hut t-SNE and Fast-Fourier interpolation based t-SNE). Results are shown to be robust across both t-SNE implementations, and all the replication attempts were successful. We additionally run the same protocol with an UMAP embedding, and observed similar robustness compared to t-SNE based algorithms. Both Linux and Windows were used during the analysis, and thus, results should be reproducible in both Operative Systems.
Randomization	All animals comprised a single group of young boars, and therefore, no further categorization was made during the study.
Blinding	Blinding was not relevant to our study, as there should not be differentiable traits (age, race...) across the animals tested, other than sperm motility features that were key for one of the goals of the study (to predict fertility).

Reporting for specific materials, systems and methods

We require information from authors about some types of materials, experimental systems and methods used in many studies. Here, indicate whether each material, system or method listed is relevant to your study. If you are not sure if a list item applies to your research, read the appropriate section before selecting a response.

Materials & experimental systems

n/a	Involved in the study	
☒	☐	Antibodies
☒	☐	Eukaryotic cell lines
☒	☐	Palaeontology and archaeology
☐	☒	Animals and other organisms
☒	☐	Human research participants
☒	☐	Clinical data
☒	☐	Dual use research of concern

Methods

n/a	Involved in the study	
☒	☐	ChIP-seq
☒	☐	Flow cytometry
☒	☐	MRI-based neuroimaging

Animals and other organisms

Policy information about studies involving animals; ARRIVE guidelines recommended for reporting animal research

Laboratory animals	The study did not involve laboratory animals.
Wild animals	The study did not involve wild animals.
Field-collected samples	The authors of the present study had no direct interaction with field animals. However, relevant data for the study (sperm motility data and boar fertility data) were retrieved from farm animals (Batallé S.A., Riudarenes, Girona). Animal housing and maintenance was performed by Batallé following standard conditions (appropriate food and water provided, animal health control,...), protocols and legislation.
Ethics oversight	No ethical approval was required in this study, as all animal-related data used in the study was provided by third parties (Batallé S.A.) who routinely follow adequate protocols for animal housing and maintenance.

Note that full information on the approval of the study protocol must also be provided in the manuscript.
